# Microbiome as a prediction of immunotherapy response in lung cancer

**DOI:** 10.3389/fimmu.2026.1849553

**Published:** 2026-07-23

**Authors:** Leonardo Rojas, Jairo Zuluaga, Andrés F. Cardona

**Affiliations:** 1 Thoracic Tumors Unit, Luis Carlos Sarmiento Angulo Cancer Treatment and Research Center-CTIC, Bogotá, Colombia; 2 GIGA/TERA Research Group, CTIC/Universidad El Bosque, Bogotá, Colombia; 3 Institute of Research and Education/Thoracic Oncology Unit, Luis Carlos Sarmiento Angulo Cancer Treatment and Research Center-CTIC, Bogotá, Colombia

**Keywords:** gut microbiome, immune checkpoint inhibitors, immunotherapy response, lung cancer, oral microbiome, predictive biomarkers

## Abstract

Immune checkpoint inhibitors (ICIs) have revolutionized the treatment of lung cancer (LC), offering durable responses in non-small cell lung cancer (NSCLC) and, to a lesser extent, small cell lung cancer (SCLC). Nevertheless, clinical outcomes remain highly heterogeneous, with many patients experiencing primary or acquired resistance and/or immune-related adverse events (irAEs) that impair their quality of life and treatment adherence. The human microbiome, particularly in the gut and oral compartments, has emerged as a critical modulator of systemic antitumor immunity and a promising noninvasive predictive biomarker for ICI efficacy and toxicity. This narrative review synthesizes the current evidence on microbiome composition, diversity, and function in patients with LC receiving ICIs as monotherapy, dual blockade, or in combination regimens, as well as clinically relevant biomarkers associated with treatment response and toxicity. Higher gut microbial alpha diversity and enrichment of beneficial taxa (e.g., *Akkermansia muciniphila, Faecalibacterium prausnitzii*, and certain *Firmicutes*) are consistently linked to improved progression-free survival (PFS) and overall survival (OS), mediated by microbial metabolites such as short-chain fatty acids and inosine, which enhance T-cell priming, tumor microenvironment remodeling, and gut-lung axis communication. Microbiome-disruptive exposures, particularly antibiotics and proton pump inhibitors (PPIs), induce dysbiosis and are strongly associated with poorer survival outcomes. Mechanistic insights from preclinical models and clinical cohorts, alongside clinical confounders, underscore the complementary role of the microbiome relative to established markers such as programmed death-ligand 1 (PD-L1) and tumor mutational burden. Prospective standardization of metagenomic profiling and microbiome-modulating interventions represents a key next step in translating these findings into personalized immunotherapy strategies for LC.

## Introduction

Immune checkpoint inhibitors (ICIs) have emerged as promising treatment options for patients with multiple cancers at different stages. Lung cancer (LC) remains the leading cause of cancer-related mortality worldwide, with non-small cell lung cancer (NSCLC) comprising approximately 85% of all cases (1). Treatment with ICIs has emerged as a promising option for monotherapy, dual immune checkpoint blockade, or in addition to chemotherapy, targeted therapy, and radiotherapy. However, marked heterogeneity in treatment response and the occurrence of immune-related adverse events (irAEs) pose significant challenges in clinical practice.

The composition and function of the microbiome exert profound effects on human health and disease, influencing immune system development, tolerance, and inflammatory responses (2). Changes in microbiome composition have been shown to affect the efficiency of various therapies, including cancer immunotherapy (3). Owing to its critical role in immune activation and modulation of cancer development and progression, the microbiome has gained considerable attention as a potential predictive biomarker and therapeutic target to enhance the success of immune checkpoint blockade.

The gut microbiome is the most extensively studied compartment in lung cancer owing to its size and systemic immune effects (2). Emerging data also suggest contributions from the oral microbiome and potential interactions with the gut-lung axis. Disruptions caused by commonly prescribed medications, such as antibiotics and proton pump inhibitors (PPIs), can further alter these microbial ecosystems and affect ICI outcomes. This review examines the current evidence regarding the gut and oral microbiomes in predicting responses to ICIs in LC, the influence of antibiotic and PPI exposure, and discusses the clinical implications, limitations, and future perspectives for microbiome-based precision immunotherapy.

## Methods

This narrative review is based on a structured but non-systematic search of PubMed/MEDLINE, Embase, and Scopus from January 2015 to April 2026, supplemented by hand-searching of reference lists and relevant congress proceedings (ASCO, ESMO, WCLC). The search terms combined were “lung cancer,” “NSCLC,” “SCLC,” “immune checkpoint inhibitor,” “immunotherapy,” “gut microbiome,” “oral microbiome,” “respiratory microbiome,” “dysbiosis,” “antibiotics,” “proton pump inhibitor,” and “predictive biomarker.” We prioritized peer-reviewed clinical cohorts and randomized data on lung cancer, complemented by mechanistic preclinical and pan-cancer studies in which lung cancer-specific evidence was absent. Reviews, editorials, and non-English publications were excluded from the study. Study selection emphasized relevance to predictive biomarker value and clinical translation rather than exhaustive coverage; no formal risk of bias or PRISMA protocol was applied.

## Gut microbiome and prediction of response of ICI in lung cancer

The gut harbors the largest microbial community in the human body, with most bacteria residing in the colon. Contrary to the frequently cited 10:1 ratio, current estimates indicate that bacterial and human cell numbers are of a similar order of magnitude (approximately 3.8x10^13 bacteria versus approximately 3.0x10^13 human cells in a 70-kg reference adult) (4). The gut microbiome contributes to immune system development, tolerance, and inflammatory regulation, and its perturbation (dysbiosis) has been associated with a range of cardiometabolic, inflammatory, and neoplastic diseases. The microbiome consists of many unicellular and multicellular organisms, such as bacteria, fungi, viruses, protozoa, and archaea, most of which are symbiotic in nature. The intestine is mainly colonized by four phyla of bacteria: *Firmicutes, Bacteroidetes, Proteobacteria*, and *Actinobacteria (*5). Alpha and beta diversity indices were used to evaluate the inter- and intra-sample relationships in the oral and gut microbiota.

Notably, virtually all available clinical evidence linking the microbiome to ICI outcomes in lung cancer is derived from NSCLC cohorts; dedicated data on SCLC are lacking. Given the distinct biology, mutational landscape, and immune contexture of SCLC, microbiome-immunotherapy associations cannot be assumed to behave similarly across lung cancer subtypes, and SCLC-specific prospective studies represent an important unmet need.

### Defining a healthy microbiome

A central aspect of this review is the relationship between chronic diseases and the microbiome. The main issues involve discussions about how to define a “healthy individual” and “dysbiosis,” emphasizing the need for precise definitions and methodologies. Central to this definition is the consideration of a wider perspective on gut health, beyond the absence of digestive complaints. A comprehensive interpretation includes the composition of the gut microbiome and its role in digestion, nutrient uptake, inflammatory modulation, and host immune status (2).

A healthy gut microbiota is characterized by its diversity, composition, and function. High bacterial diversity contributes to homeostasis, enhances resistance to external disturbances, and promotes rapid recovery. Reduced diversity has been linked to cardiometabolic diseases and cancer (5–7). High consumption of animal protein and fat and low fiber consumption are associated with higher mucosal proliferative biomarkers, namely, CD3+ intraepithelial lymphocytes and CD68+ lamina propria macrophages. Moreover, they are associated with microbiota-mediated colon cancer by increasing saccharolytic fermentation and butyrogenesis and suppressing secondary bile acid synthesis (8).

### Gut microbiome and immunotherapy toxicity

Gastrointestinal irAEs, particularly ICI-associated colitis, are among the most frequent immune toxicities observed. These symptoms are the second-most reported irAEs with ICIs and typically develop within 6–8 weeks of starting treatment (9, 10), thereby impairing the quality of life of patients and treatment adherence.

Microbial tryptophan catabolites engage the aryl hydrocarbon receptor (AhR) and modulate intestinal immune homeostasis (11, 12). For example, indole-3-carboxaldehyde protects against ICI-induced colitis in mice without impairing antitumor activity (13). Consistent with the microbiome–toxicity axis, Andrews et al. demonstrated in a cohort of patients receiving dual ICI therapy that those who developed grade ≥3 toxicity exhibited significantly higher abundance of *Bacteroides intestinalis* and upregulation of mucosal IL-1β (14–19). These findings support the potential of microbiome profiling as a tool for identifying patients at a higher risk of severe irAEs (20).

### Gut microbiome and immunotherapy response

The composition of the gut microbiota influences the clinical benefits of ICI in patients with cancer. **Table 1** lists the main trials that explored the relationship between the gut microbiome and response to ICIs. The taxa *Firmicutes* (phylum), *Clostridiales* (order), and *Akkermansia muciniphila* have been shown to be positively correlated with clinical response and/or toxicities in the context of PD-1/CTLA-4 blockade — a correlation described primarily in melanoma cohorts by Gopalakrishnan et al. and Matson et al., with NSCLC-specific validation derived from a subgroup analysis in Routy et al. (25–27). Similarly, Xu et al. analyzed the differences in intestinal microbiota composition between 50 NSCLC patients who responded to ICIs and those who did not. There were significant differences in the microbial composition. *Firmicutes, Actinobacteria*, and *Fusobacteria* were more abundant in patients who achieved clinical benefits lasting at least six months, an observation reported in pan-cancer cohorts with a predominant representation of melanoma and gastrointestinal tumors and limited NSCLC-specific data (21).

**Table 1:** Summary of studies evaluating the gut microbiome and its association with response to immune checkpoint inhibitor (ICI) therapy in patients with non-small cell lung cancer (NSCLC).

Study (year)	Sample size/population	Disease stage	Treatment setting/line	ICI regimen	Microbiome assessment (sample type)	Timing of sampling	Sequencing platform	Clinical endpoint	Key findings — gut microbiome	Publication type
Xu Hua et al. (21)	50 patients with advanced NSCLC	Advanced/metastatic	1st-line or later (NR)	NR (anti-PD-1/PD-L1 based)	Stool	Pre-treatment	16S rRNA amplicon sequencing	Objective response/clinical benefit	*Bifidobacterium* and *Lactobacillus* abundance was higher in ICI responders; *Bacteroides* abundance was lower in ICI responders.	Full publication
Zhang F et al. (22)	48 patients with NSCLC	Stage IVA–IVB	1st-line (NR)	NR (anti-PD-1/PD-L1 based)	Stool	Pre-treatment	16S rRNA amplicon sequencing	Clinicopathological features correlation	Gut microbiota composition in NSCLC was correlated with clinicopathological features, including tumor stage and histology.	Full publication
Shoji Fumihiro et al. (23)	28 patients with NSCLC	Stage IIA–IVB	Perioperative/adjuvant (NR)	NR (anti-PD-1/PD-L1 based)	Stool	Pre-treatment	16S rRNA amplicon sequencing	Objective response/clinical benefit	Higher gut alpha-diversity and *Blautia* enrichment in responders; RF32 (unclassified genus) enriched in non-responders.	Full publication
Derosa Lisa et al. (24)	148 patients with advanced NSCLC	Advanced/metastatic	1st-line or later (NR)	NR (anti-PD-1/PD-L1 based)	Stool	Pre-treatment	Whole-genome shotgun sequencing (WGS)	ICI response/immunoresistance dynamics	TOPOSCORE, a gut microbiome-derived composite score, robustly predicts immunoresistance dynamics across cancer types.	Full publication
Andrews Miles et al. (20)	77 patients with advanced NSCLC	Advanced/metastatic	1st-line or later (NR)	NR (anti-PD-1/PD-L1 based)	Stool	Pre-treatment	Whole-genome shotgun sequencing (WGS)	Immune-related adverse events (irAEs) — grade ≥3 toxicity	Patients with grade 3 irAEs showed significantly higher *Bacteroides intestinalis* abundance and upregulation of mucosal IL-1β.	Full publication

ICI, immune checkpoint inhibitor; NSCLC, non-small cell lung cancer; NR, not reported; WGS, whole-genome shotgun sequencing; irAE, immune-related adverse event; PD-1, programmed cell death protein 1; PD-L1, programmed death ligand 1.

Based on these findings, *Faecalibacterium* may represent an important factor associated with clinical benefits in patients with melanoma treated with ICI, although this association has not been consistently replicated in lung cancer cohorts and cannot be directly extrapolated to NSCLC without further disease-specific evidence. Shoji et al. also found that “maintaining” gut microbiota diversity and a composition enriched in genus *Blautia* during ICI therapy with a mean number of 307 days ICI initiation and sample collection was associated with longer progression-free survival (PFS) patients with NSCLC (23). No consensus exists on gut fingerprinting to predict immunoresistance. TOPOSCORE, derived from shotgun metagenomics, classified 148 patients with advanced NSCLC treated with ICI alone or with chemotherapy. The eubiotic SIG2+ signature was associated with significantly longer OS than the dysbiotic SIG1+ signature (HR 0.50, 95% CI 0.36-0.71, P = 0.0001). However, this evidence is derived largely from a single development cohort; prospective, externally validated, and assay-standardized confirmation is required before TOPOSCORE-type composite scores can inform clinical decisions. This illustrates a broader gap: most gut signatures are promising but lack the multicenter prospective validation needed for biomarker qualification (24).

Emerging evidence suggests that gut microbiota composition may affect distal tumor lesions. In murine models, anti-CTLA-4 blocking monoclonal antibodies (mAbs) have been shown to induce dysbiosis, increase bacterial translocation, stimulate IL-12 secretion by dendritic cells (DCs), and prime commensal-specific Th1 cells capable of recognizing tumor cells through antigen mimicry. Although this mechanistic framework is well established in preclinical systems, its direct clinical relevance in NSCLC has not yet been demonstrated and requires prospective validation in lung cancer cohorts (28). In addition, other specific bacteria, most notably Bifidobacterium, originally characterized in murine melanoma models by Sivan et al., have been associated with increased DC maturation, enhanced CD8+ T cell priming, and accumulation within the tumor microenvironment (TME), findings that have not been specifically replicated in NSCLC (29).

**Figure 1:**
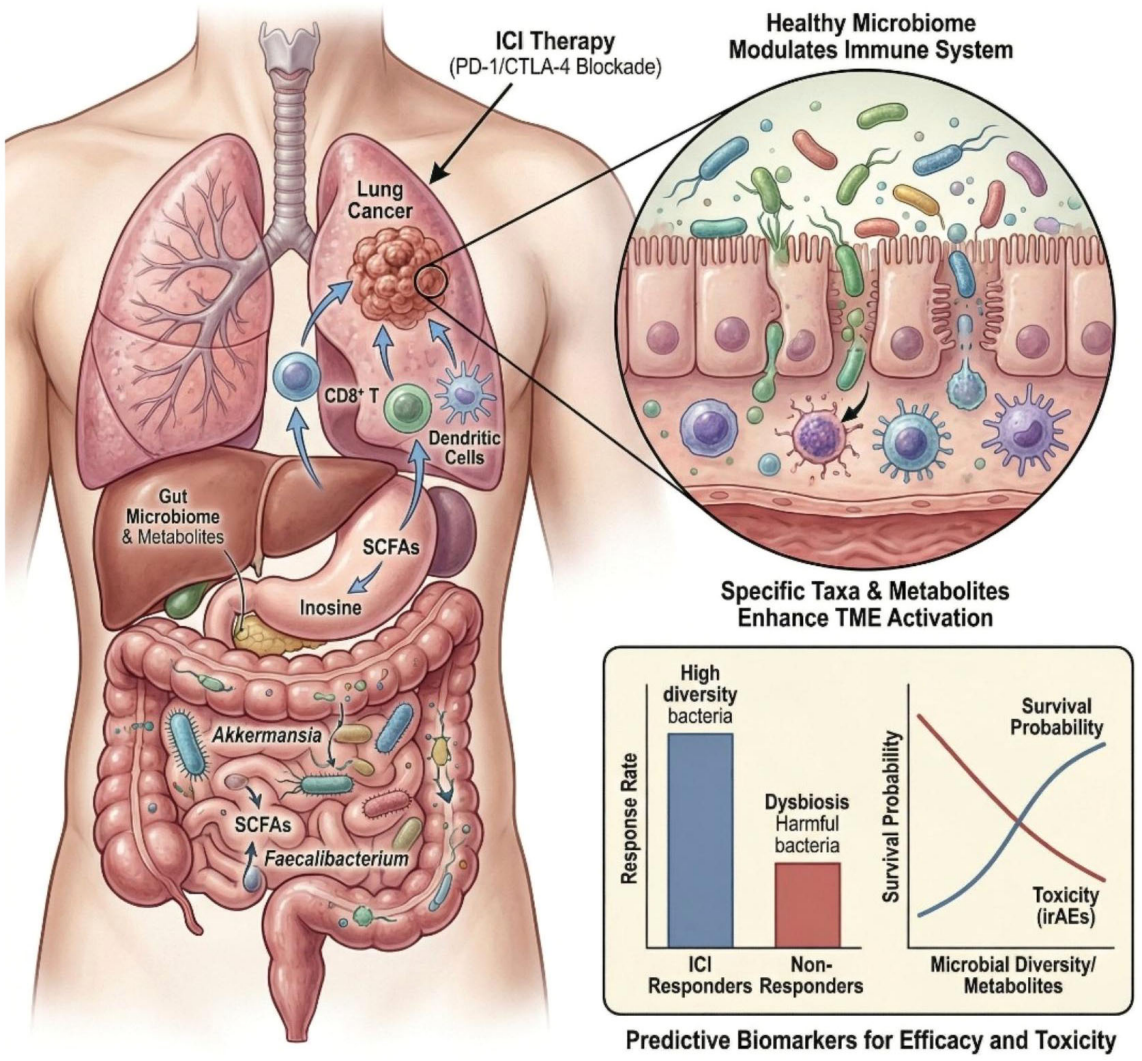
Gut microbiome composition, microbial metabolites, and immune axis as predictors of immune checkpoint inhibitor (ICI) response and toxicity in lung cancer. The gut-lung axis is depicted centrally: commensal bacteria—including Akkermansia muciniphila and Faecalibacterium prausnitzii—produce SCFAs and inosine that promote CD8^+^ T-cell and DC activation, enhancing antitumor immunity at the lung tumor site under PD-1/CTLA-4 blockade. The inset shows a diverse intestinal epithelium supporting immunological homeostasis and TME activation. Summary panels illustrate higher ICI response rates with greater gut microbial alpha-diversity (vs. dysbiosis) and a concurrent reduction in irAE burden as microbial diversity increases. ICI, immune checkpoint inhibitor; PD-1, programmed cell death protein 1; CTLA-4, cytotoxic T-lymphocyte-associated protein 4; CD8^+^ T, CD8-positive cytotoxic T lymphocyte; DC, dendritic cell; SCFA, short-chain fatty acid; TME, tumor microenvironment; irAE, immune-related adverse event; NSCLC, non-small cell lung cancer. Data sources: Routy B et al. Science. 2018;359:91–97; Derosa L et al. Nat Med. 2022;28:315–324; Shoji F et al. Front Mol Biosci. 2022;9:1040424; Coutzac C et al. Nat Commun. 2020;11:2168; Andrews MC et al. Nat Med. 2021;27:1432–1441; Xu H et al. Heliyon. 2024;10:e29899.

The activation of immune cells within the TME has become a key element in increasing the efficacy of ICIs. In addition to the direct effects of commensal bacteria, certain bacterial groups produce metabolites with immunogenic properties. Urolithin A (Uro A), short-chain fatty acids (SCFAs), lipopolysaccharide (LPS), and inosine act on diverse components of the TME, reshaping its immune landscape. In pancreatic adenocarcinoma murine models,Uro A a natural compound produced by the commensal gut microbiome from foods rich in ellagitannins exhibited a dual positive effect: it downregulated the PI3K/AKT/mTOR oncogenic pathway, reduced tumor burden, and attenuated immunosuppressive tumor-associated M2-like macrophages while concurrently increasing infiltration of CD4+ and CD8+ T cells with a memory-like phenotype and downregulating PD-L1 expression (30–32). In contrast to these findings, Denk et al. reported that differential changes in CD4^+^ populations may vary across solid tumor types (33). Short-chain fatty acids (SCFAs), mainly acetate (C2), propionate (C3), and butyrate (C4), are produced in the colon through bacterial fermentation of dietary fiber. Butyrate exerts systemic anti-inflammatory functions by reducing immune cell migration, adhesion, and cytokine expression and modulating cellular processes, such as proliferation and apoptosis (34). Coutzac et al. elegantly demonstrated, in patients with advanced melanoma treated with ipilimumab and in parallel murine experimients, that butyrate and propionate levels may exert detrimental effects on anti-CTLA-4 activity by decreasing tumor-specific and memory T cells and while boosting the proportion of regulatory T cells (Tregs) (35); whether analogous SCFA-mediated effects occur in patients with NSCLC receiving anti-PD-1 agents has not been directly evaluated. Microbial metabolites can modulate cytokine networks, thereby influencing the therapeutic activity of ICIs. Bacterial LPS elevates interleukin (IL)-6 production, promoting tumor cell proliferation through activation of the Janus kinase/signal transducer and activator of transcription (JAK/STAT) pathway, a mechanism characterized in preclinical models and cell lines across multiple tumor types; however, direct evidence in NSCLC remains to be established (36). Within the TME, elevated IL-6 levels not only attenuate the antitumor efficacy of ICIs but also exacerbate immune-related adverse events (irAEs) (37). In contrast, interleukin-12 (IL-12) exerts beneficial effects by reprogramming the TME; it directly engages IL-12 receptors on CD8^+^ T cells to enhance their activation, facilitates CD4^+^ T-cell infiltration, and suppresses regulatory T-cell (Treg) populations, ultimately improving ICI outcomes (38, 39). Multiple microbial metabolites regulate IL-12 secretion; LPS stimulates its release, whereas butyrate inhibits it (40). **Figure 1** (Gut microbiome composition, microbial metabolites, and immune axis as predictors of immune checkpoint inhibitor (ICI) response and toxicity in lung cancer) summarizes the relationship between the gut microbiome and ICI response.

Toward predictive application, candidate features can be grouped into three tiers: (i) taxonomic markers (favorable: Akkermansia muciniphila, Faecalibacterium prausnitzii, and Bifidobacterium spp.; unfavorable: Bacteroides dorei and intratumoral Fusobacterium); (ii) functional/metabolic markers (short-chain fatty acid biosynthesis pathway abundance, inosine, and tryptophan-AhR metabolites), which preliminary data suggest may outperform taxonomy alone; and (iii) composite clinical-microbial scores, such as TOPOSCORE. Validating these as biomarkers will require shotgun metagenomic profiling for strain- and function-level resolution, supervised machine-learning models (e.g., LASSO-regularized regression, random forests, gradient boosting) trained and tested with nested cross-validation, reporting of discrimination (AUC, Harrell c-index), and calibration, and, critically, prospective external validation in independent NSCLC cohorts with rigorous adjustment for antibiotics, PPIs, diet, smoking, and performance status.

## Oral/respiratory microbiome and immunotherapy response in lung cancer

Direct evidence from oral and airway microbiome studies remains limited, and predictive analyses of the buccal microbiome have not been reported. **Table 2** lists the main trials that explored the relationship between the oral microbiome and the ICI response.

**Table 2:** Summary of studies evaluating the oral microbiome and its association with response to immune checkpoint inhibitor (ICI) therapy in patients with non-small cell lung cancer (NSCLC).

Study (year)	Sample size/population	Disease stage	Treatment setting/line	ICI regimen	Microbiome assessment (sample type)	Timing of sampling	Sequencing platform	Clinical endpoint	Key findings — oral microbiome	Publication type
Dan Hui Huang et al. (83)	20 patients with NSCLC	Stage IIIB–IV	1st-line or later (NR)	NR (anti-PD-1/PD-L1 based)	Saliva	Pre-treatment	Metagenomic NGS (shotgun)	Objective response; PD-L1 expression correlation	Several *Neisseria* and *Actinomyces* species were enriched in treatment responders, and salivary bacterial signatures were strongly correlated with PD-L1 expression.	Full publication
Augustyn et al. (41)	53 patients with locally advanced NSCLC	Locally advanced (stage III)	Concurrent chemoimmunotherapy or chemoradiation	Anti-PD-1/PD-L1 (chemoimmunotherapy arm)	Buccal swabs; stool	Pre-treatment	16S rRNA amplicon sequencing	PFS, DFS, OS	Higher buccal microbiome diversity was associated with significantly improved PFS, DFS, and OS. This predictive value was specific to patients who received chemoimmunotherapy and not chemoradiation alone. [Verify HR and direction against primary source.]	Conference abstract (preliminary/unpublished)
Zapata-García et al. (42)	55 patients with NSCLC	Stage III–IV	1st-line or later (NR)	NR (anti-PD-1/PD-L1 based)	Saliva; stool	Pre-treatment	16S rDNA amplicon sequencing	Objective response	No significant differences in oral/salivary alpha or beta diversity were observed between the responders and non-responders.	Full publication
Chau et al. (43)	16–34 patients with advanced or metastatic lung cancer	Advanced/metastatic	1st-line or later (NR)	NR (anti-PD-1/PD-L1 based)	Buccal, nasal, and fecal samples	Pre-treatment and longitudinal	16S rRNA amplicon sequencing	Objective response; irAEs	This prospective study examined the correlation between oral and nasal microbiome composition and ICI response, as well as the development of immune-related adverse events. [Reference 83 removed; cite as ref. 84 only.]	Full publication
Shoji Fumihiro et al. (23)	28 patients with NSCLC	Stage IIA–IV	Perioperative/adjuvant (NR)	NR (anti-PD-1/PD-L1 based)	Saliva; fecal samples	Pre-treatment	16S rRNA amplicon sequencing	Objective response/clinical benefit	No significant differences were observed in the oral or salivary microbiome composition between ICI responders and non-responders, highlighting the limited predictive utility of the oral microbiome in this cohort.	Full publication

ICI, immune checkpoint inhibitor; NSCLC, non-small cell lung cancer; NR, not reported; NGS, next-generation sequencing; irAE, immune-related adverse event; PD-1, programmed cell death protein 1; PD-L1, programmed death ligand 1; PFS, progression-free survival; DFS, disease-free survival; OS, overall survival.

Chau et al. collected buccal, nasal, and fecal samples from patients with lung cancer treated with anti-PD-1/PD-L1 agents and revealed alterations in gut communities and associations between gut taxa, toxicity, and response (43). The MicroDurva trial (conference abstract; results unpublished), which enrolled patients with unresectable stage III NSCLC on durvalumab maintenance, included longitudinal buccal sampling with documentation of antibiotic, probiotic, and PPI exposure (22).

A systematic review analyzing oral microorganisms in saliva, sputum, bronchoalveolar lavage fluid (BAL), and tumor tissue from patients with lung cancer suggested that *Veillonella* may improve ICI response (44); however, the predictive contribution of the oral compartment was not isolated in this study.

**Figure 2:**
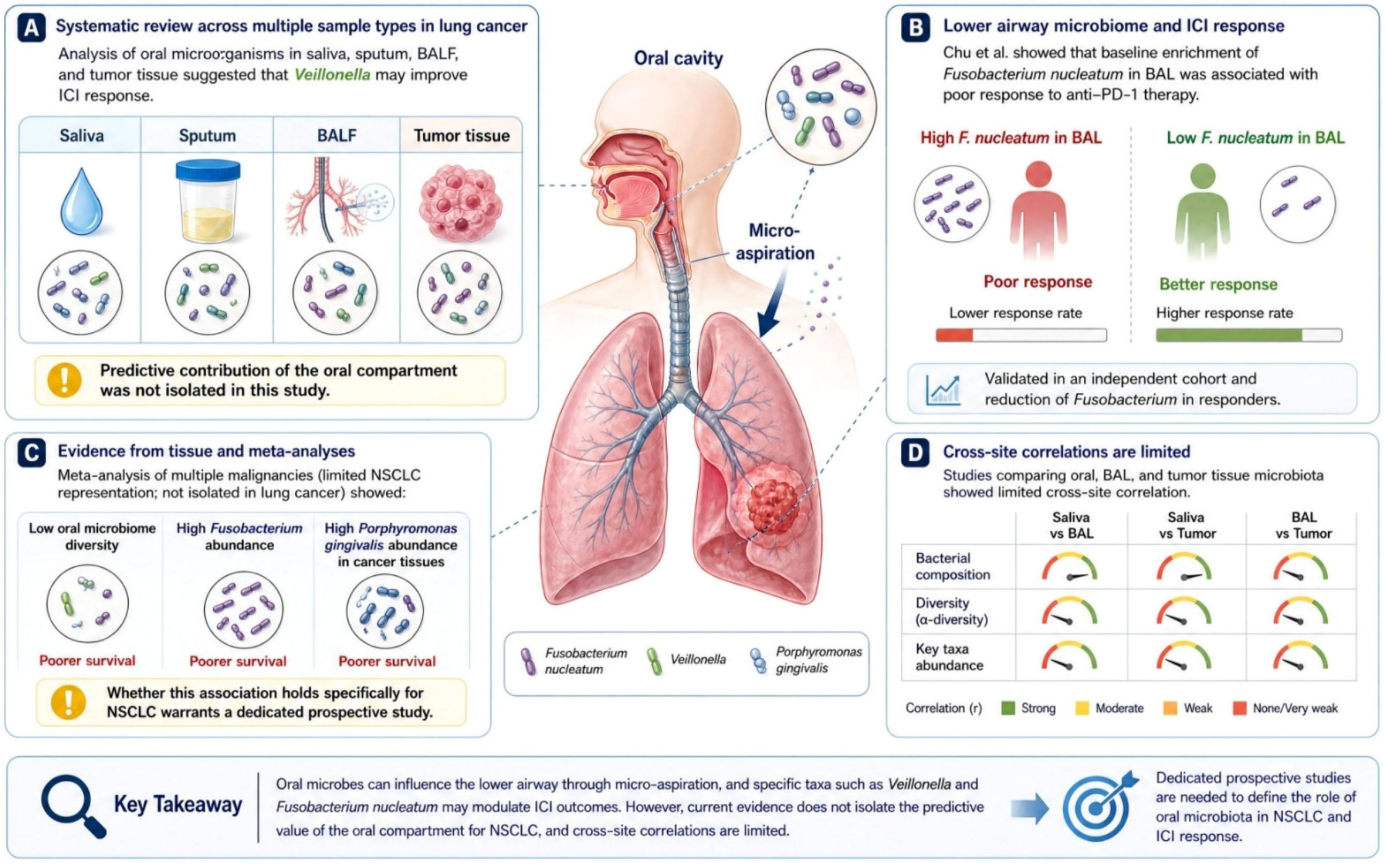
Oral and lower airway microbiota in non-small cell lung cancer and ICI response: insights and gaps from current evidence. **(A)** Systematic review of oral microorganisms (saliva, sputum, BALF, tumor tissue) identified Veillonella as potentially associated with improved ICI response; the independent predictive contribution of the oral compartment was not isolated. **(B)** Chu et al. showed that baseline enrichment of Fusobacterium nucleatum in BAL was independently associated with poor anti-PD-1 response, validated in an independent cohort. **(C)** Meta-analyses (limited NSCLC representation) show that low oral alpha-diversity and high Fusobacterium/Porphyromonas gingivalis abundance correlate with poorer survival; NSCLC-specific prospective validation is warranted. **(D)** Cross-site correlations (saliva vs. BAL vs. tumor) for bacterial composition, alpha-diversity, and key taxa are predominantly weak to absent, indicating oral communities do not reliably reflect lower airway microbiome status. The central panel illustrates the micro-aspiration pathway connecting oral and lower airway compartments. NSCLC, non-small cell lung cancer; ICI, immune checkpoint inhibitor; PD-1, programmed cell death protein 1; BALF, bronchoalveolar lavage fluid; BAL, bronchoalveolar lavage; irAE, immune-related adverse event; r, Pearson or Spearman correlation coefficient. Data sources: Chu S et al. Onco Targets Ther. 2022;15:201–213; Kwiatkowska AM et al. Transl Oncol. 2025;62:102557; Li S et al. Syst Rev. 2024;13:41; Zhu B et al. Front Microbiomes. 2025;4:1589686; Zapata-García M et al. Heliyon. 2024;10:e33684; Padure A et al. Microorganisms. 2025;13:1565.

Related evidence comes from studies of the lower airway microbiome, which shares taxa with the oral cavity through micro-aspiration. Chu et al. demonstrated that baseline enrichment of *Fusobacterium nucleatum* in BAL was associated with poor response to anti-PD-1 therapy, with validation in an independent cohort and reduction of *Fusobacterium* in responders (45). A meta-analysis of multiple malignancies with limited NSCLC-specific representation and without isolated evaluation in lung cancer found that low oral microbiome diversity and high abundance of *Fusobacterium* or *Porphyromonas gingivalis* in cancer tissues were correlated with poorer overall, disease-specific, and progression-related survival (46). However, whether this association holds specifically for NSCLC warrants a dedicated prospective study. Cross-site correlations between oral, BAL, and lung tissue microbiota were limited, suggesting that oral communities may not fully reflect the lower airway niches involved in ICI mechanisms. **Figure 2** (Oral and lower airway microbiota in non-small cell lung cancer and ICI response: insights and gaps from current evidence) summarizes the relationship between the oral and respiratory microbiomes and the response to ICIs.

### Oral microbiome dynamics during cancer therapy

Studies have investigated the impact of cancer treatments on the oral microbiome. A pilot cohort study of patients undergoing chemotherapy or chemoimmunotherapy reported significant reductions in oral microbial diversity post-treatment, with taxonomic shifts based on the treatment type (46). Chemotherapy alone was associated with increased abundance of *Streptococcus, Gemella*, and *Granulicatella* and decreased abundance of *Neisseria* and *Veillonella* species. Combined chemoimmunotherapy led to an increase in *Streptococcus* and a reduction in *Haemophilus* and *Neisseria* (47). These findings suggest that immunotherapy with cytotoxic agents may exert selective pressure on oral microbial communities. However, the limited sample size precludes definitive conclusions. Broader oncological data have linked oral dysbiosis to adverse outcomes. A meta-analysis of multiple malignancies found that low oral microbiome diversity and high abundance of *Fusobacterium* or *Porphyromonas gingivalis* in cancer tissues were correlated with poorer overall, disease-specific, and progression-related survival (48). This reinforces the notion that oral microbiome perturbations can influence systemic oncological trajectories beyond the lungs. Interventional studies using oral-targeted approaches, such as miraculin-containing supplements in cancer patients with dysgeusia, have documented oral dysbiosis, characterized by reduced diversity and taxon-specific shifts (49). However, these changes have not yet been linked to the performance of ICIs.

### Antibiotic and proton pump inhibitor exposure

Clinical evidence linking the microbiome to ICI outcomes in NSCLC stems from analyses of antibiotic and PPI exposure as indicators of microbial disruption. However, these studies do not differentiate between oral and gut effects. Meta-analyses and pooled randomized data show that antibiotic use around ICI therapy initiation is associated with poorer survival outcomes, with pooled hazard ratios for OS ranging from 1.3 to 2.0 and PFS from 1.2 to 1.5 (50–54). The adverse effect is strongest when antibiotics are administered within 30–60 days before or after ICI initiation, during immune priming, and early effector response, whereas later antibiotic use shows variable associations (52–54). Although some single-center cohorts have shown no significant impact, aggregated evidence supports the crucial role of microbiome integrity in achieving optimal ICI efficacy in NSCLC (55, 56).

PPIs provide indirect evidence by altering gut communities through oral commensal translocation (57). Pooled analyses of NSCLC trials indicate that concurrent PPI use is associated with shorter PFS and OS in patients receiving atezolizumab but not docetaxel, suggesting an immunotherapy-specific interaction (58). A pan-cancer meta-analysis of ICI-treated patients — encompassing multiple tumor types beyond NSCLC, with a magnitude of effect that may differ by cancer type — reports consistent negative associations between proton pump inhibitor (PPI) exposure and survival outcomes, further implicating microbiome-mediated mechanisms (57); NSCLC-specific estimates from dedicated analyses are needed before these findings can be applied uniformly to lung cancer practice.

### Broader biomarker context

In the broader biomarker landscape, the microbiome occupies an adjunctive position alongside established markers such as PD-L1 and tumor mutational burden (TMB). A meta-analysis suggested that multimodal biomarker models and high TMB outperform PD-L1 immunohistochemistry in predicting response to ICIs, whereas microbiome-based classifiers provide incremental gains of borderline significance (59). Emerging data show that microbiota-associated predictors may operate independently of PD-L1 expression and that tumor or gut microbial diversity can identify responders among patients with low TMB, highlighting the complementarity between genomic and microbial markers (57, 60).

## Discussion

This narrative review synthesizes the available evidence on the microbiome as a predictive biomarker of response to immune checkpoint inhibitors in lung cancer. Understanding the factors associated with ICI response and toxicity is crucial for optimizing the treatment outcomes. Baseline patient characteristics, pathological features, and concurrent therapies are related to changes in the gut microbiota and response to ICIs. Zhang et al. reported differences in gut microbiota composition according to smoking history, NSCLC histology and clinical stage (61). In addition, common therapeutic interventions, such as antibiotics and PPI exposure, are associated with low ICI efficacy and significantly impair OS, PFS, and ORR. In contrast, probiotic supplementation has demonstrated promising results by synergistically adding value to patients receiving ICIs (62).

Our review consolidates evidence from multiple independent cohorts, illustrating that the gut microbiome has a markedly stronger and more consistent predictive capacity for PFS in patients with LC undergoing treatment with ICIs than the oral and respiratory microbiomes (23, 41, 63).

Elevated baseline alpha diversity within the gut serves as a robust indicator of extended PFS and favorable clinical outcomes across geographically diverse populations (23, 63, 64). These observations position the gut microbiome as one of the most promising non-invasive biomarkers currently under investigation in immuno-oncology research.

Taxonomic signatures within the gut further stratify patient outcomes; however, their effects are highly context-dependent (54). Specific taxa, such as *Akkermansia muciniphila, Bifidobacterium* species, and *Faecalibacterium prausnitzii*, have consistently emerged as beneficial bacteria associated with enhanced ICI efficacy (65, 66). Notably, the predictive value of *Akkermansia* abundance was dose-dependent. While moderate abundance is advantageous, overdominance, particularly following antibiotic exposure, correlates with an immunosuppressive environment and diminished PFS (65, 67). Conversely, taxa such as *Bacteroides dorei, Dialister*, and certain *Streptococcus* species are frequently enriched in non-responders (68–70). Findings regarding *Prevotella copri* were divergent across Asian and Western cohorts, reflecting the significant influence of dietary habits and microbial metabolic versatility (71). This setting-specific effect underscores the fact that microbial biomarkers cannot be assumed to perform uniformly across different disease stages and treatment regimens.

However, the overall quality of evidence remains modest. Most available studies are retrospective or single-center with relatively small sample sizes. Methodological heterogeneity is substantial, particularly regarding sequencing platforms (16S rRNA amplicon sequencing versus shotgun metagenomics), sample collection timing, and bioinformatic pipelines. These factors limit the reproducibility and generalizability of the reported microbial signatures. Furthermore, most studies have not adequately adjusted for important clinical confounders, such as antibiotic exposure, proton pump inhibitor use, smoking status, performance status, and dietary habits, all of which can independently influence both microbiome composition and clinical outcomes. Consequently, while associations between gut microbiome features and ICI response have been frequently reported, causal inference remains limited.

In stark contrast to the gut, the oral and respiratory microbiomes have demonstrated limited and inconsistent prognostic utility (23, 72). Most studies evaluating salivary or oral microbiomes have found no significant differences in diversity between ICI responders and non-responders (23). A critical exception was identified based on the treatment regimen: buccal microbiome diversity successfully predicted improved PFS exclusively in patients receiving chemoimmunotherapy combinations rather than ICI monotherapy (41). Importantly, the predictive value of the oral/buccal microbiome appears to be setting- and regimen-dependent. The single positive signal (higher buccal diversity associated with improved PFS) was observed in locally advanced NSCLC treated with chemoimmunotherapy, not in metastatic disease receiving ICI monotherapy, where salivary/oral profiles showed no significant discrimination between responders and non-responders (23, 42, 64). This regimen dependence argues against the generalization of oral microbiome biomarkers to the metastatic ICI-monotherapy setting. Furthermore, the intratumoral microbiome represents a distinct predictive compartment in which local microbial diversity and specific taxa, such as Fusobacterium, are independently associated with resistance to immune checkpoint blockade (73). A central theme emerging from this evidence is that functional metabolic capacity is a more reliable and consistent predictor of PFS than taxonomic composition alone (74). Machine learning models incorporating functional pathways systematically outperformed simple taxonomic profiles (73). Specifically, the enrichment of SCFA production pathways, including butyrate and propionate, is consistently correlated with extended survival, irrespective of geographic or dietary differences (75). This underscores the mechanistic role of bacterial metabolites in modulating systemic immunity, enhancing the infiltration of effector T cells into the TME, and facilitating communication along the gut-lung axis (76).

The relationship between microbiome composition and ICI efficacy is significantly influenced by clinical confounders, particularly antibiotic and PPIs use. The administration of antibiotics prior to or concurrently with the initiation of ICIs markedly reduces microbial diversity, leading to dysbiotic states that substantially impair PFS and OS (74, 77). Similarly, PPIs alter the gut microbiome, resulting in unfavorable overrepresentation of oral pathobionts (78).

The clinical application of these findings requires a shift from single-species biomarkers to composite scores that integrate multiple bacterial taxa, functional profiles and clinical parameters. As evidenced by large-scale validations, incorporating microbiome signatures with variables such as cachexia, antibiotic history, and treatment modalities has resulted in highly accurate predictive models (79).

These findings raise several questions with important clinical implications. Will routine microbiome sequencing provide reliable predictive biomarkers? Most studies are either retrospective or single-center with small sample sizes, and we need to consider this question in larger cohorts and across different cancer types. Second, should microbiome-modifying factors be systematically monitored in patients treated with ICIs? Increasing evidence demonstrates that patients who receive antibiotics immediately before or after ICI initiation have poorer outcomes. Finally, can we manipulate the gut and oral microbiota to enhance the responses? As demonstrated by Routy et al. and others, the microbiome can be modulated using several strategies, including fecal microbiota transplantation and the administration of live bacterial products (27, 80).

Future research should prioritize standardized methodologies, particularly the transition from 16S rRNA to whole-metagenome shotgun sequencing, to accurately capture functional pathways and strain-level variations (23, 80). Profiling the gut microbiome offers a profound non-invasive strategy for predicting immunotherapy responses in LC and actively modulating the host immune axis to overcome resistance.

### Microbiome-modulating strategies to overcome resistance.

In addition to its role as a predictive biomarker, the microbiome is an actionable therapeutic target. It is important to distinguish this evidence, which is largely translational and early phase, from the predictive associations discussed above. Strategies include dietary fiber, defined probiotics (e.g., *Clostridium butyricum*), live bacterial products, and fecal microbiota transplantation (FMT) (81). These interventions are hypothesis-generating and have not yet been validated for routine clinical use. Recent prospective lung cancer-specific data reinforce this rationale. Huang et al. reported on FMT for advanced NSCLC with secondary PD-1 resistance, with microbiome-diversity correlates of benefit (82). In the multicenter, open-label phase 2 FMT-LUMINate trial, a single oral healthy donor FMT prior to first-line pembrolizumab in PD-L1-high NSCLC achieved an objective response rate of 80% (16/20) with a disease-control rate of 95% and no grade ≥ 3 adverse events, and benefit appeared to depend on depletion of specific deleterious taxa rather than simple engraftment (83). A separate single-arm exploratory study combining healthy donor FMT with anti-PD-1 rechallenge in previously treated NSCLC was feasible and tolerable, with preliminary clinical activity (84). These early-phase results support FMT as a biologically active immunomodulatory strategy while underscoring the need for randomized confirmation.

## Conclusions

In conclusion, these findings indicate that the gut microbiome is the strongest and most consistent predictor of immunotherapy response in lung cancer, whereas the oral microbiota shows a biologically plausible but less established association. However, a fundamental discrepancy exists between the specificity of the clinical question—whether baseline oral microbiome composition can predict ICI response in lung cancer—and the current evidence, which focuses on gut or airway studies and indirect exposure surrogates. To address this gap, future research should prioritize adequately powered prospective cohorts of ICI-treated NSCLC and SCLC patients, incorporating standardized oral sampling at baseline, parallel gut and airway profiling, shotgun metagenomic sequencing with functional pathway analysis, and rigorous adjustment for antibiotic and PPI use, smoking status, performance status, and treatment regimens. Such designs are essential to determine whether oral microbiome signatures offer independent or additive predictive value beyond established clinical and molecular factors in lung cancer.
